# Reply to Lakhan and Wicks: Placement of drug–software combinations within VISTA’s platform taxonomy

**DOI:** 10.1073/pnas.2522921122

**Published:** 2025-09-19

**Authors:** Charles H. Jones

**Affiliations:** ^a^Independent Researcher, Mansfield, TX 76063

We thank Lakhan and Wicks for highlighting how pairing prescription digital therapeutics with medicines (“software-enhanced drugs”) could accelerate development and value creation (1). Our article delineated two platform classes—molecule-enabled and design-enabled—and operationalized value using the VISTA framework across Strategic, Technical, and Adaptive domains with explicit metrics and implementation guidance (Fig. 1, p. 4; Table 2, pp. 6-8) (2). Within this taxonomy, drug–software combinations are not a third, orthogonal class; they are a design-enabled expression and, when codeveloped atop a pharmacologic platform, a hybrid (“dual-platform”) configuration that VISTA already scores.

Regulatory pathways, including FDA’s draft Prescription Drug Use–Related Software (PDURS) guidance and the Software as a Medical Device (SaMD) construct, clarify whether software is integrated with a drug, a combination product, or regulated independently (3, 4). VISTA is pathway agnostic: It evaluates value so long as claims, controls, and update processes are specified. As Lakhan and Wicks note, exemplars are emerging (e.g., CT-132 for episodic migraine prevention) that make such integrations increasingly concrete (1).

A concise VISTA mapping follows (2). Strategic: apply the Revenue Diversification Index (RDI) to bundled indications/geographies; extend the IP Leadership Score (IPLS) to software copyrights, algorithmic IP, and data use rights; and use the Partnership Strength Index (PSI) for collaborations with DTx firms, provider systems, and payers. Technical: parameterize Probability of Technical Success (PTS) with additive effects beyond convenience (e.g., adherence-adjusted exposure, validated digital endpoints, decision–support accuracy) and capture software update cadence and traceability within cycle time efficiency (CTE); and retain the manufacturing efficiency ratio (MER) for the drug component. Adaptive: Innovation Velocity Index (IVI) captures rapid content/indication releases; talent leverage ratio (TLR) captures cross-functional teams spanning psychology, product, and data science; ecosystem evolution score (EES) quantifies learning with health systems and payers.

We share the authors’ optimism but underscore evidentiary standards befitting drug claims. Demonstrating pharmacologic–software synergy requires randomized or well-controlled quasi-experimental designs that isolate effects beyond engagement. Labeling should specify what improves (exposure, persistence, clinical endpoints) and for whom. For earlier programs, VISTA still provides decision value by anchoring to analogous benchmarks and tracking leading indicators such as engagement durability, release-governance/traceability, and early safety-signal detection.

In sum, software-enhanced drugs fit naturally within design-enabled platforms and, in many settings, hybrid dual-platform systems. The VISTA framework already accommodates this modality; the refinements above enable comparative scoring alongside traditional and platform-based approaches. We appreciate Lakhan and Wicks for prompting this clarification and look forward to empirical applications (1, 2).
